# The Role of the Human Immune System in the Aging Process: a Mathematical Model of Cell and Cytokine Activation

**DOI:** 10.1093/geroni/igab046.2546

**Published:** 2021-12-17

**Authors:** Kian Talaei, Steven Garan, Nuno Martins, Joshua Cho, Julia Jahansooz, Puneet Bhullar, Elliott Suen, Walter Piszker

**Affiliations:** 1 University of California, Berkeley, Center for Research and Education in Aging, University of California, Berkeley, California, United States; 2 University of California, Berkeley, Berkeley, California, United States; 3 Mayo Clinic, Scottsdale, Arizona, United States; 4 UC Berkeley, Berkeley, California, United States

## Abstract

The role of the human immune system as a factor in the aging process has led to extensive research in the field of infection biology and bioinformatics. Cell-based mathematical models have previously been used to simulate the immune system in response to pathogens. A variety of cells, such as activated and resting macrophages, plasma cells, antibodies, helper T cells, T-lymphocytes, and B-lymphocytes, have already been simulated by mathematical models. This work aims to incorporate cytokines in these mathematical models to create a more comprehensive simulation that can predict cytokine levels in response to a Gram-positive bacterium, S. aureus. To accomplish this, the cytokines Tumor Necrosis Factor Alpha (TNF-α), Interleukin 6 (IL-6), Interleukin 8 (IL-8), and Interleukin 10 (IL-10) were studied to quantify the relationship between cytokine release from macrophages and the concentration of the pathogen, S. aureus ex vivo. The results of the simulation were compared to ex vivo human whole blood data to test its accuracy. The future expansion of this model may provide a clearer image of the various interactions within the immune system and this improved model of the immune system may also facilitate a better understanding of the mechanisms that lead to the degradation of the immune system during the aging process.

